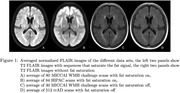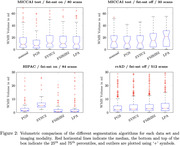# Comparison of MRI T2 FLAIR White Matter Hyperintensity Segmentation Algorithms in Clinical Trials

**DOI:** 10.1002/alz.089535

**Published:** 2025-01-09

**Authors:** Norman Scheel, Josh Hubert, Joshua H. Baker, Zachary Fernandez, Pavel G. Yanev, Ann M Stowe, Wanpen Vongpatanasin, Rong Zhang, David C Zhu

**Affiliations:** ^1^ Michigan State University, East Lansing, MI USA; ^2^ University of Kentucky, Lexington, KY USA; ^3^ UT Southwestern Medical Center, Dallas, TX USA

## Abstract

**Background:**

White Matter Hyperintensities (WMH) appear on T2‐weighted Fluid‐Attenuated Inversion Recovery (FLAIR) Magnetic Resonance Imaging (MRI) and are an important biomarker of cerebral small vessel disease (CSVD), cognitive decline, and stroke. However, manual delineation is laborious and bias‐prone, while automated segmentation has proven challenging. With the recent conclusion of the MICCAI‐Society WMH segmentation challenge, and our large clinical trials, “Risk Reduction for Alzheimer’s Disease” (rrAD) and “Hypertension, Intracranial Pulsatility and Brain Amyloid‐beta Clearance in older adults” (HIPAC), we investigated the differences in the total WMH volume segmented by various algorithms to ensure the extraction of accurate and meaningful image‐derived phenotypes.

**Methods:**

The rrAD trial provides 512 2D axial T2 FLAIR scans, fat saturation off (ages 60‐85). The HIPAC study provides 84 3D T2 FLAIR scans, fat saturation on (ages 55‐79). The MICCAI WMH challenge provides a test set of 110 scans of 2D axial T2 FLAIR images, 30 without, and 80 with fat saturation. Our clinical trials and the MICCAI datasets comprise data from over 700 subjects, across 10 scanners and diverse sequences. Three of the best‐performing MICCAI algorithms were selected: PGS, Sysu‐Media‐2, FMRIB‐TrUE‐Net2, as well as the LPA algorithm from SPM. Segmentation results were randomized, and fully blinded expert raters scored a subset of 120 scans.

**Results:**

Figure 1 depicts the average normalized images for each T2 FLAIR contrast. The MICCAI test set shows a median WMH burden of 10‐17 ml, while rrAD subjects presented with relative medians of ≈3−4 ml, and HIPAC subjects with ≈2−3 ml. Figure 2 shows the WMH volume for each segmentation algorithm and dataset. For HIPAC Sysu‐Media‐2 had high false pickup within grey matter regions, while LPA produced high false pickup at the posterior septum pellucidum. PGS scored best in manual ratings.

**Conclusions:**

MRI sequences that use fat saturation change the T2 FLAIR image contrast substantially and impact segmentation performance, we observed better performance with fat saturation off. The MICCAI challenge algorithms outperformed classical methods (PGS leading), detecting small lesions, away from the ventricles. As WMH distribution, shape, and longitudinal tracking are likely more sensitive biomarkers than volume alone, further analysis is warranted.